# Benchmark for multi-cellular segmentation of bright field microscopy images

**DOI:** 10.1186/1471-2105-14-319

**Published:** 2013-11-07

**Authors:** Assaf Zaritsky, Nathan Manor, Lior Wolf, Eshel Ben-Jacob, Ilan Tsarfaty

**Affiliations:** 1 Blavatnik School of Computer Science, Tel Aviv University, Tel Aviv, 69978, Israel; 2 School of Physics and Astronomy, The Raymond and Beverly Sackler Faculty of Exact Sciences, Tel Aviv University, Tel-Aviv, 69978, Israel; 3 Center for Theoretical Biological Physics, Rice University, Houston, TX, 77005-1827, USA; 4 Research & Development Unit Assaf Harofeh Medical Center, Zerifin, 70300, Israel; 5 Department of Clinical Microbiology and Immunology, Sackler School of Medicine, Tel Aviv University, Tel Aviv, 69978, Israel

**Keywords:** Collective cell migration, Wound healing assay, Segmentation, Benchmarking

## Abstract

**Background:**

Multi-cellular segmentation of bright field microscopy images is an essential computational step when quantifying collective migration of cells in vitro. Despite the availability of various tools and algorithms, no publicly available benchmark has been proposed for evaluation and comparison between the different alternatives.

**Description:**

A uniform framework is presented to benchmark algorithms for multi-cellular segmentation in bright field microscopy images. A freely available set of 171 manually segmented images from diverse origins was partitioned into 8 datasets and evaluated on three leading designated tools.

**Conclusions:**

The presented benchmark resource for evaluating segmentation algorithms of bright field images is the first public annotated dataset for this purpose. This annotated dataset of diverse examples allows fair evaluations and comparisons of future segmentation methods. Scientists are encouraged to assess new algorithms on this benchmark, and to contribute additional annotated datasets.

## Background

Characterizing and quantifying collective migration phenotypes of a monolayer of cells in vitro is an important step in understanding physiological processes such as development, wound repair and cancer motility. The prevalent approach is to acquire still or time-lapse images using bright field microscopy, followed by manual or automated extraction of quantitative measures of cellular morphology or dynamics (e.g., [1-3]).

The vast numbers of microscopic images acquired in high throughput studies preclude manual annotation and hence automatic computational tools become indispensable. Indeed, several tools to tackle these tasks were recently reported; some exploit local motion-estimation to quantify dynamic intercellular phenomena [4,5], whereas others are designed to quantify only global motion of complete colonies or confluent monolayers [6-15]. The basic common computational step in all approaches is segmentation of an image into cellular and non-cellular regions, the accuracy of which is crucial for further analysis. It is inherently a foreground-background segmentation task: no explicit cell segmentation is performed; each pixel is rather assigned a binary label as being part of either a cellular or a non-cellular region.

The high variability in imaging conditions and cells’ appearance requires robust algorithms that can deal with this imaging diversity automatically, accurately and preferably without the need for parameter-tuning. It is difficult to systematically select the most appropriate segmentation tool from the available options [16,17]. Proposed methods are usually evaluated on in-house benchmarks that are not freely available to the public. These evaluations often compare accuracy to human-annotations and rarely to alternative computational methods, hence are not subjected to a thorough comparative assessment of extant methods [18].

We therefore propose a uniform framework to benchmark algorithms for multi-cellular segmentation in bright field microscopy images.

## Construction and content

**Figure 1:**
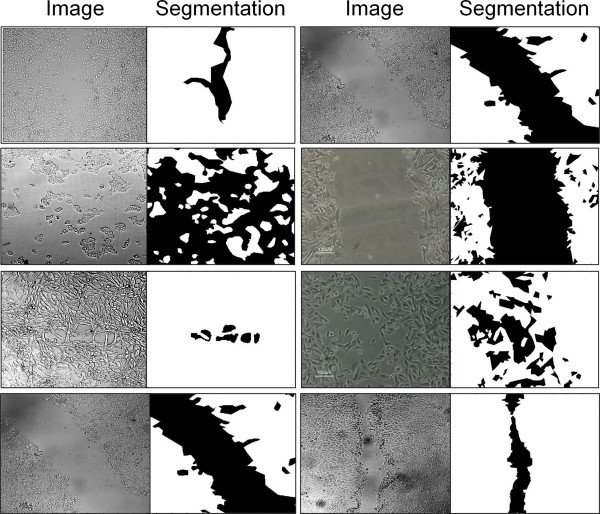
Examples of images from the presented benchmark and their corresponding manual segmentations.

A set of 171 manually segmented images of 5 different cell lines at diverse confluence levels, acquired in several laboratories under different imaging conditions, were partitioned into 8 datasets as follows (example images are presented in Figure 1, detailed description of the cells and imaging conditions can be found on the benchmark website):

• *TScratch*: 24 bright field images of confluent cells available at the TScratch site, http://www.cse-lab.ethz.ch/index.php?&option=com_content&view=article&id=363[6];

• *Melanoma*: 20 bright field images of confluent populations of brain metastatic melanoma cells acquired during a wound healing experiment [19];

• *Init*: 28 differential interference contrast (DIC) images of confluent DA3 cells, derived from the mouse mammary adenocarcinoma line D1-DMBA-3, acquired during wound healing experiments;

• *SN15*: 54 DIC images of confluent DA3 cells acquired during a multi-well wound healing experiment;

• *Scatter*: 6 DIC images of Madin-Darby Canine Kidney (MDCK) epithelial cells acquired during a multi-well scatter experiment (unpublished data);

• *Microfluidics*: 13 DIC images of MDCK cells grown in a microfluidic plate acquired during a scatter assay experiment with a Hepatocyte growth factor/scatter factor gradient (unpublished data);

• *HEK293*: 12 DIC images of confluent HEK293T cells acquired during a multi-well wound healing experiment (unpublished data);

• *MDCK*: 14 DIC images of confluent MDCK cells acquired in a multi-well wound healing experiment (unpublished data).

Four out of eight datasets (“TScratch”, “Melanoma”, “Init”, “SN15”) were previously reported before in our study comparing TScratch to MultiCellSeg [15]. The other four are published here for the first time. “TScratch” is the only dataset that was already publically available.

Two freely available tools (TScratch, [6]; MultiCellSeg, [15]) and one implementation of a designated algorithm [10] were evaluated on these datasets; a brief description of these algorithms is found in the Additional file 1: Supporting Text. Each of the algorithms was evaluated using the same set of parameters on all datasets to assess robustness. All raw images, manual segmentations, algorithm segmentation results, performance measurements and an evaluation Matlab script are freely available at the Broad Bioimage Benchmark Collection (BBBC, http://www.broadinstitute.org/bbbc/) [20].

The algorithms were ranked by mean F-measure (i.e., the harmonic mean of precision and recall) of the pixel labeled across all images in each dataset $\left(\frac{1}{n}\sum_{i=1}^{n}\text{fmeasure}\left(\text{Imag}e_{i}\right)\right)$, where n is the number of images in the given dataset). F-measure is the evaluation measure used for foreground-background datasets in the BBBC. Evaluation results are found in Table 1, which also present the median F-measure as a more robust (less sensitive) evaluation. Additional file 2: Table S1 displays the average precision and recall measures, Additional file 3: Figure S1 plots the performance on each of the images in each dataset.

**Table 1:** Evaluation of the three designated tools on the eight available datasets

**Algorithm/Dataset**	**Init**	**SN15**	**Melanoma**	**TScratch**	**Scatter**	**Microfluidics**	**HEK293**	**MDCK**
**Mean F-Measure**	**(N = 28)**	**(N = 54)**	**(N = 20)**	**(N = 24)**	**(N = 6)**	**(N = 13)**	**(N = 12)**	**(N = 14)**
**(Median F-Measure)**								
**[F-Measure Adjusted]**								
Tscratch (Geback et al. 2009)	0.96	0.96	0.88	**0.94**	0.47	0.42	0.90	0.92
	(0.96)	(0.97)	(0.90)	(0.93)	(0.47)	(0.41)	(0.91)	(0.93)
MultiCellSeg (Zaritsky et al. 2011)	**0.98**	**0.97**	0.85	0.93	0.55	0.35	**0.95**	**0.96**
	(0.98)	(0.98)	(0.91)	(0.95)	(0.56)	(0.45)	(0.95)	(0.98)
Topman et al. 2011	**0.98**	0.95	**0.93**	0.78	**0.58**	**0.63**	0.85	0.89
	(0.98)	(0.97)	(0.93)	(0.76)	(0.60)	(0.63)	(0.87)	(0.93)
	[0.97]	[0.96]	[0.93]	[0.84]	[0.52]	[0.61]	[0.84]	[0.93]

F-measure was used for evaluation in three forms: mean F-measure of images in the dataset, median, and mean after threshold adjustment on the training set (for [10]).

Best mean F-measure performance is marked in bold.

To assess the baseline variance that should be expected when scoring the results of an algorithm, a second expert annotated an arbitrary partial set of the images (64 images from all datasets, excluding the “Scatter” dataset). The two annotations were evaluated using mean and median F-measures compared with the primary annotated ground truth. The results are reported in Additional file 4: Table S2, and in the Additional file 1: Supporting Text. The annotators were instructed to pay special attention to small isolated cells and voids between groups of cells; this is important for some applications and can be later excluded via post-processing if not relevant. Most inconsistencies found were in defining the exact border contours of the cells, as described in the Additional file 1: Supporting Text and in Additional file 5: Figure S2.

Twenty arbitrary images were selected as a training set for algorithms that apply supervised learning [15], or for adjusting parameters’ values. Use of different arbitrarily selected training images did not significantly change the algorithms’ performance. Comprehensive assessment of [10] is presented in Additional file 6: Table S3 and discussed in the Additional file 1: Supporting Text; Table 1 contains the optimal results achieved considering a single set of parameters for all datasets.

## Utility

The benchmark includes two directories:

• train: 20 images (images directory) and the corresponding manual annotations (manual directory). These images can be used as a training set for algorithms that apply supervised learning or for adjusting parameters’ values.

• datasets: 8 different datasets, each consists of images (images directory), ground truth manual annotations (manual directory), results masks of the 3 algorithms we compare (tscratch, multiCellSeg, topman directories), a measures file (measures.mat) containing evaluation summary, and a second annotation for a partial set of the images.

∘ A documented Matlab script (bbbcCalcMeasures.m) that compares the different algorithms (and can easily be adjusted for comparing new algorithms).

Benchmark structure is described in the README file in the main directory.

## Discussion

The wound healing assay (aka scratch assay), the traditional method used to study collective cell motility and migration [21,22] in the life sciences, is performed by inducing a sudden injury created by removal of a sheet of cells from a confluent monolayer [19]. This assay can be performed using multi-well plates, with up to 384-wells [23], providing a large amount of data for high-quality quantitative analysis. The scratch is imaged and measured periodically during the healing process, and rate of change in the wound area is recorded and can be compared with other cell lines, environmental conditions or chemical treatments. Quantifying wound healing assays is a natural application of multi-cellular segmentation algorithms. The availability of a benchmark to evaluate algorithms on a variety of cell lines and imaging conditions will enable educated algorithm selection. The general segmentation of cell clusters in bright-field images has additional applications (e.g., quantifying scatter assays [15]), thus emphasizing the importance of evaluating the segmentation of non-confluent cells images. The dataset provided is diverse in terms of cell lines, image acquisition parameters, cellular confluence levels, and was collected from several laboratories, and can thus address the need for public access to image repositories [24] as well as the general concern regarding poor algorithmic comparisons [17,18,25,26].

## Conclusions

A variety of software tools and imaging apparatuses exist to enable high throughput multi-cellular segmentation in bright field images. This is the first and currently only freely available public annotated dataset for evaluations. We encourage scientists to evaluate new algorithms and to contribute additional annotated datasets to this benchmark.

## Availability and requirements

All raw images, manual segmentations and evaluations are freely available at the Broad Bioimage Benchmark Collection (BBBC), http://www.broadinstitute.org/bbbc/BBBC019/.

## Competing interests

The authors declare that they have no competing interests.

## Authors’ contributions

AZ collected the data, performed the manual segmentation, evaluated the algorithms, published the data sets and wrote the manuscript. NM performed the second annotation and the ground truth evaluation. NM, LW, EBJ and IT revised the manuscript. All authors read and approved the final manuscript.

## Supplementary Material

Additional file 1 **Supporting Text.** This file contains a brief description of the evaluated algorithms, notes on parameter tuning, details on evaluation of Topman’s thresholding method, and details on assessing the baseline variance in the annotated data. Click here for file

Additional file 2: Table S1 Precision/recall. Precision/recall of all algorithms on all datasets. Click here for file

Additional file 3: Figure S1 Direct comparison of algorithms on all images. Image-by-image evaluation. Scatter plots displaying for each image the F-measure produced by the 3 algorithms. Each x-axis entry represents an image (ordered by the filename), y-axis is the F-measure. Red – Tscratch, Green – MultiCellSeg, Cyan – Topman’s algorithm. **a,** Init. **b,** NN15. **c,** Melanoma. **d,** TScratch. **e,** Scatter. **f,** Microfluidics. **g,** HEK293. **h,** MDCK. Click here for file

Additional file 4: Table S2 Baseline variance. An arbitrary partial set of the images (62 images from all datasets, excluding the “Scatter” dataset) was selected to be annotated by another expert. This annotation was compared with the primary annotated ground truth by calculating the mean F-measure to assess the baseline variance of each dataset. Click here for file

Additional file 5: Figure S2 Baseline variance examples. Visualization of inconsistencies between manual annotations by different experts. Annotations shown were selected from the dataset with higher baseline variance (“Melanoma”, “Miscrofluidics”). The green channel is the raw image, the blue channel is the official annotation of cells, and the red channel is the second annotation. Thus, light-magenta represents agreement in annotation of cells, green represents agreement in annotation of non-cellular regions, light-red represents regions annotated as non-cellular in the ground truth but as cellular by the second expert, light blue represents regions that were annotated as cellular according to the ground truth but non-cellular according to the second expert. It is clear from this visualization that most inconsistencies appear at cell borders. Click here for file

Additional file 6: Table S3 Adjusting Tompan’s algorithm. The automatic threshold extraction method in Topman’s algorithm was evaluated compared to a constant threshold. Evaluation of different values demonstrated that a constant threshold surpasses the automatic adjustment for most datasets. The best value found was used to evaluate this algorithm’s performance in the main text. Click here for file
