# Supplementary material for: Benchmark for multi-cellular segmentation of bright field microscopy images
Source: BMC Bioinformatics. 2013 Nov 7;14:319. doi: 10.1186/1471-2105-14-319 (PMC3826518; doi:10.1186/1471-2105-14-319)
Supplement: Additional file 1 — Supporting Text. This file contains a brief description of the evaluated algorithms, notes on parameter tuning, details on evaluation of Topman’s thresholding method, and details on assessing the baseline variance in the annotated data. [file 1471-2105-14-319-S1.pdf]

Benchmark for Multi-Cellular Segmentation of Bright Field Microscopy Images – Supporting Text

Assaf Zaritsky[1*], Nathan Manor[1], Lior Wolf[1], Eshel Ben-Jacob[2,3,4] and Ilan Tsarfaty[5,*]

[1] Blavatnik School of Computer Science, Tel Aviv University, Tel Aviv 69978, Israel.

[2] School of Physics and Astronomy, The Raymond and Beverly Sackler Faculty of Exact Sciences, Tel Aviv University, Tel Aviv 69978, Israel.

[3] Center for Theoretical Biological Physics, Rice University, Houston, TX 77005-1827, USA.

[4] Research & Development Unit Assaf Harofeh Medical Center , Zerifin, 70300, Israel.

[5] Department of Clinical Microbiology and Immunology, Sackler School of Medicine, Tel Aviv University, Tel Aviv 69978, Israel.

[*] To whom correspondence should be sent: Ilan Tsarfaty, E-mail: ilants@post.tau.ac.il; Assaf Zaritsky, E-mail: assafzar@gmail.com

Brief description of the evaluated algorithms: TScratch [1] is freely available software that uses fast discrete curvelet transform [2] to segment and measure the area occupied by cells in an image. The curvelet transform extracts gradient information in several scales, orientations and positions in a given image, and encodes it as curvelet coefficients. TScratch selects two scale levels to fit the gradient details found in cells contours, and combines the two scale levels to generate a curvelet magnitude image, which incorporates the details of the original image in the selected scales. Morphological operators are further applied to refine the curvelet magnitude image. As a final step, an automatic threshold is applied to partition the curvelet magnitude image into occupied and free regions. This approach was first applied for edge detection in microscopy images [3]. TScratch can be used via a GUI or by applying the source code which are available together with a detailed user manual at http://www.cse-lab.ethz.ch/index.php?&option=com_content&view=article&id=363. This is by far the most developed software of the three options, featuring a convenient user interface, the ability to set several parameters and compatibility with most platforms.

Topman *et al.* [4] used standard deviation of pixel intensities as a measure for texture. It is calculated across two different scales for every given image, producing an image of texture homogeneities for each scale. A threshold based on the smaller scale is automatically defined, as half the highest local maximum of pixel intensities in the histogram of the texture image. This threshold is applied to segment the texture images of both scales. The final segmentation is calculated as the intersection of these two segmented masks followed by morphological operators. Source code is available in [4].

MultiCellSeg [5] is a freely software available that applies machine-learning based classification to local patches within an image. Basic image features are used to represent each patch which is fed to a designated pre-train support vector machine [6] (using LIBSVM implementation [7]). Post processing includes additional classification of larger connected components and graph-cut segmentation to reclassify erroneous regions and refine the segmentation. A basic GUI and the source code are available at http://www.cs.tau.ac.il/~assafzar/. A main feature is that MultiCellSeg requires no parameter tuning as the classifiers are computed based on the training images. However, this is the slowest of the three algorithms presented here.

Parameter tuning: RGB images were converted to gray level images by the built-in Matlab function rgb2gray for all algorithms. The set of parameters used for each algorithm were set once and then used to compare the algorithms' performance across all 8 datasets. MultiCellSeg is parameter-free so no parameters were set. The default set of parameters was used to evaluate TScratch. The algorithm by Topman *et al.* [4] was evaluated using scales of 9 and 25 pixels to fit the values reported in the paper; small changes of these values had minor effect on the algorithm's performance. Additional attention was given to the thresholding method as described below.

Extensive evaluation of Topman's thresholding: While examining Topman's algorithm, we discovered that the automatic threshold extraction method performed inferiorly compared to a constant threshold. Thus, we evaluated the performance of different values for this parameter. The best performance for 6 of the 8 datasets was achieved at a threshold value of 0.03. These results are summarized in Additional file 6: Table S3. Next, we tried to set the threshold parameter based on the training images. The threshold value was selected to optimize the average F-measure of the images in the training set, then applied to the test datasets. The performance was similar to the results obtained before, see Table 1 for details.

Assessing the baseline variance in annotation: A second expert annotated a partial set of arbitrary images from 7 out of the 8 datasets (excluding Scatter, which has only 6 images). To assess the baseline variance created by different human annotations we used the average F-measure to compare between the second annotation and the official ground truth annotation, in the same manner as used when comparing the different algorithms. Four datasets ("Init", "SN15", "HEK293", and "MDCK") achieved an agreement F-measure of 0.98 or higher, TScratch had a median F-measure of 0.95. To retain a better confidence on the remaining two high-variance datasets, a second annotation was performed on all images in these sets. "Melanoma" median F-measure was 0.93 while "Microfluidics" was 0.81. Indeed, the performance of all algorithms on "Microfluidics" was significantly inferior compared with the other datasets (Table 1). Example visualizations of the agreements and inconsistencies between the two annotations on images from the less-consistent datasets are presented in Additional file 5: Figure S2, we conclude that datasets of scattered cells have higher baseline variance since F-measure is sensitive to inconsistent annotations at cell borders (The pros for using F-measure are its simplicity and sensitivity; when the score is high – annotations are definitely very similar). Additional file 4: Table S2 summarizes all baseline variance results. The second annotation is also provided as part of the benchmark.

References

1. Geback T, Schulz MM, Koumoutsakos P, Detmar M: **TScratch: a novel and simple software tool for automated analysis of monolayer wound healing assays**. *Biotechniques* 2009, **46**(4):265-274.
2. Candes E, Demanet L, Donoho D, Ying LX: **Fast discrete curvelet transforms**. *Multiscale Modeling & Simulation* 2006, **5**(3):861-899.
3. Geback T, Koumoutsakos P: **Edge detection in microscopy images using curvelets**. *BMC Bioinformatics* 2009, **10**:75.
4. Topman G, Sharabani-Yosef O, Gefen A: **A standardized objective method for continuously measuring the kinematics of cultures covering a mechanically damaged site**. *Med Eng Phys* 2011.
5. Zaritsky A, Natan S, Horev J, Hecht I, Wolf L, Ben-Jacob E, Tsarfaty I: **Cell Motility Dynamics: A Novel Segmentation Algorithm to Quantify Multi-Cellular Bright Field Microscopy Images**. *Plos One* 2011, **6**(11).
6. Cortes C, Vapnik V: **Support-Vector Networks**. *Machine Learning* 1995, **20**(3):273-297.
7. Chang C-C, Lin C-J: **LIBSVM : a library for support vector machines**. In.; 2001.
