# Supplementary material for: Benchmark for multi-cellular segmentation of bright field microscopy images
Source: BMC Bioinformatics. 2013 Nov 7;14:319. doi: 10.1186/1471-2105-14-319 (PMC3826518; doi:10.1186/1471-2105-14-319)
Supplement: Additional file 2: Table S1 — Precision/recall. Precision/recall of all algorithms on all datasets. [file 1471-2105-14-319-S2.pptx]

## Slide 1
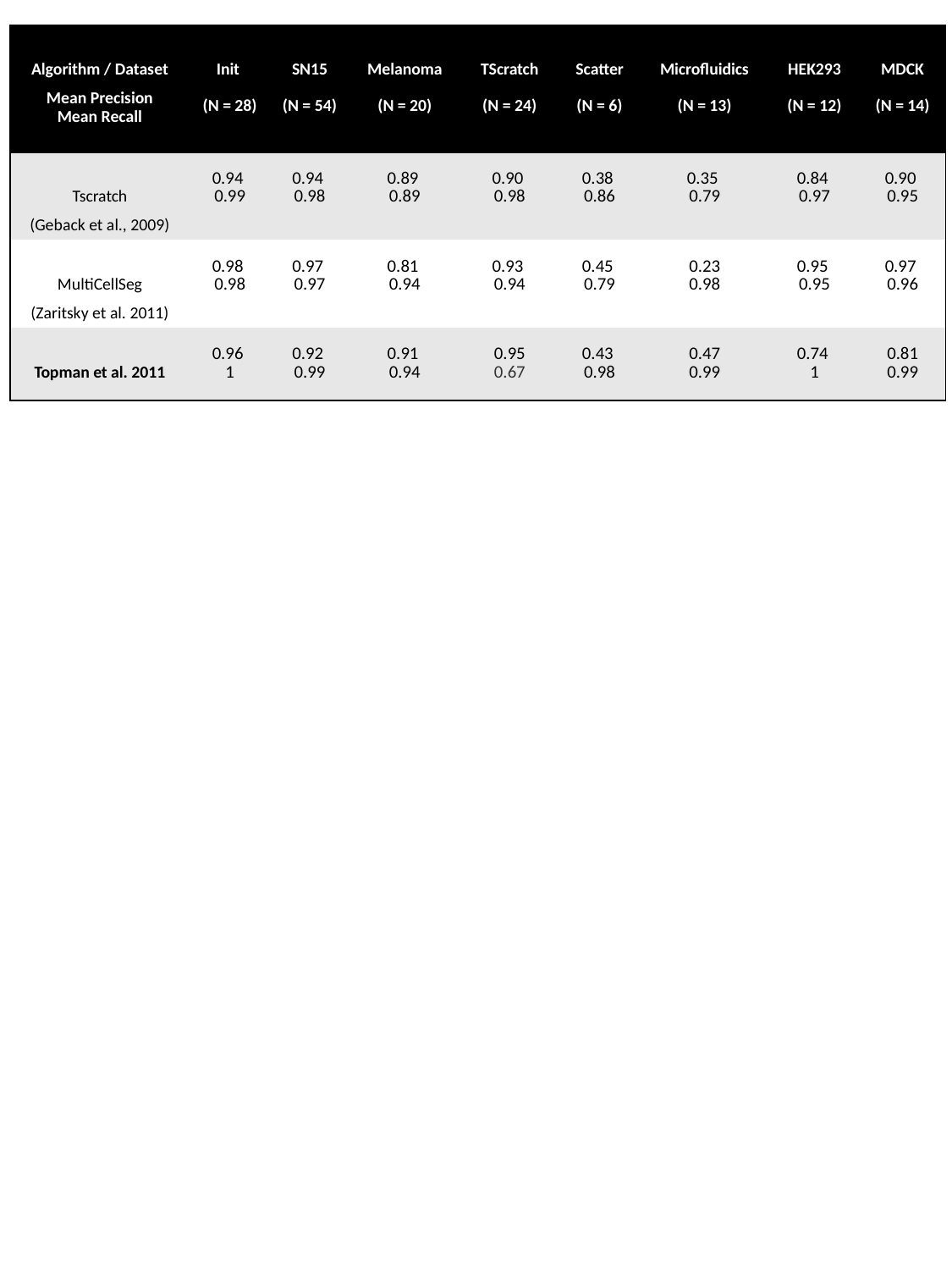

| Algorithm / Dataset Mean Precision Mean Recall | Init (N = 28) | SN15 (N = 54) | Melanoma (N = 20) | TScratch (N = 24) | Scatter (N = 6) | Microfluidics (N = 13) | HEK293 (N = 12) | MDCK (N = 14) |
| --- | --- | --- | --- | --- | --- | --- | --- | --- |
| Tscratch (Geback et al., 2009) | 0.94 0.99 | 0.94 0.98 | 0.89 0.89 | 0.90 0.98 | 0.38 0.86 | 0.35 0.79 | 0.84 0.97 | 0.90 0.95 |
| MultiCellSeg (Zaritsky et al. 2011) | 0.98 0.98 | 0.97 0.97 | 0.81 0.94 | 0.93 0.94 | 0.45 0.79 | 0.23 0.98 | 0.95 0.95 | 0.97 0.96 |
| Topman et al. 2011 | 0.96 1 | 0.92 0.99 | 0.91 0.94 | 0.95 0.67 | 0.43 0.98 | 0.47 0.99 | 0.74 1 | 0.81 0.99 |
