# Supplementary figures and images for: Benchmark for multi-cellular segmentation of bright field microscopy images
Source: BMC Bioinformatics. 2013 Nov 7;14:319. doi: 10.1186/1471-2105-14-319 (PMC3826518; doi:10.1186/1471-2105-14-319)

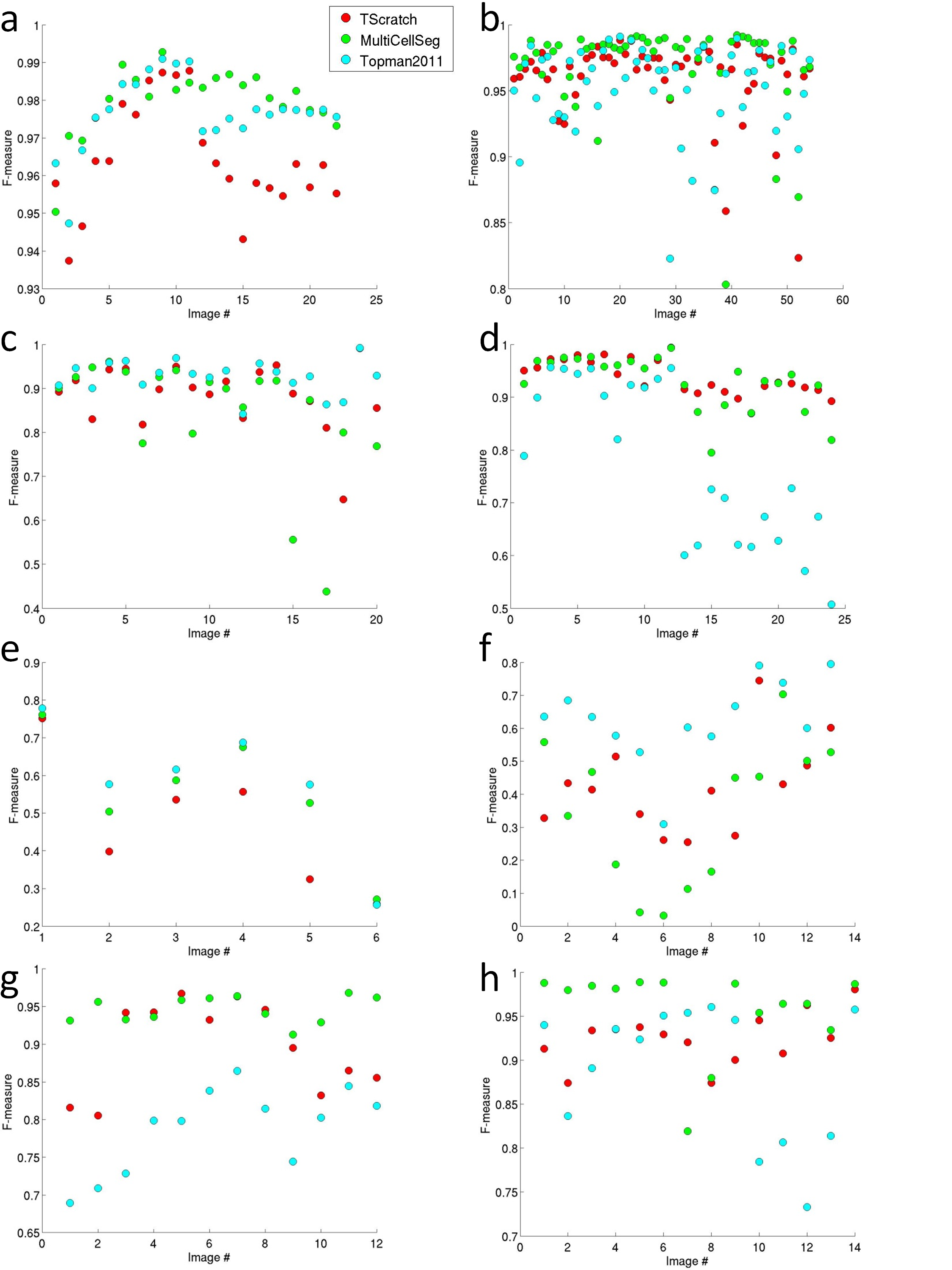

Supplement: Additional file 3: Figure S1 — Direct comparison of algorithms on all images. Image-by-image evaluation. Scatter plots displaying for each image the F-measure produced by the 3 algorithms. Each x-axis entry represents an image (ordered by the filename), y-axis is the F-measure. Red – Tscratch, Green – MultiCellSeg, Cyan – Topman’s algorithm. a, Init. b, NN15. c, Melanoma. d, TScratch. e, Scatter. f, Microfluidics. g, HEK293. h, MDCK. [file 1471-2105-14-319-S3.tiff]

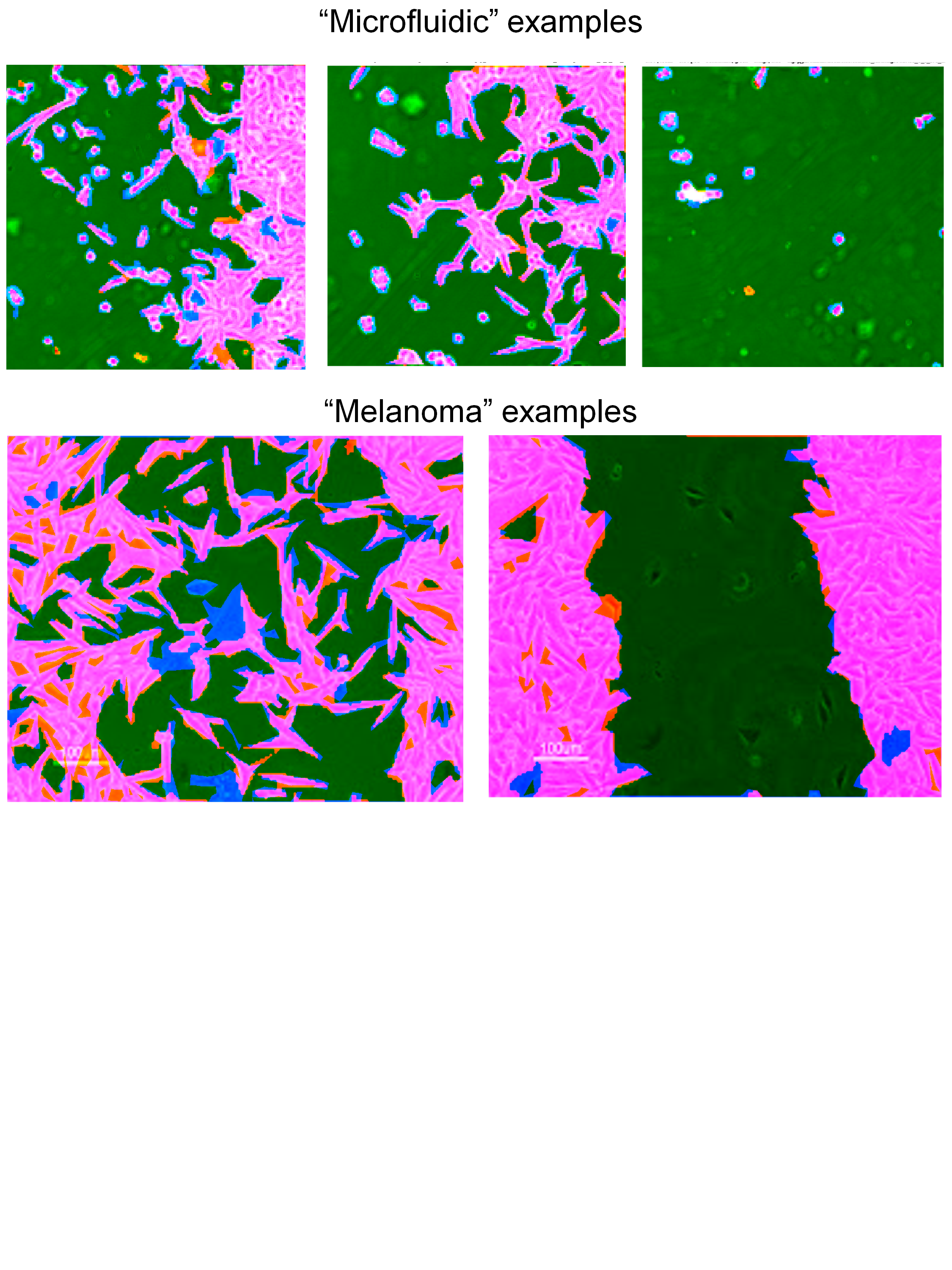

Supplement: Additional file 5: Figure S2 — Baseline variance examples. Visualization of inconsistencies between manual annotations by different experts. Annotations shown were selected from the dataset with higher baseline variance (“Melanoma”, “Miscrofluidics”). The green channel is the raw image, the blue channel is the official annotation of cells, and the red channel is the second annotation. Thus, light-magenta represents agreement in annotation of cells, green represents agreement in annotation of non-cellular regions, light-red represents regions annotated as non-cellular in the ground truth but as cellular by the second expert, light blue represents regions that were annotated as cellular according to the ground truth but non-cellular according to the second expert. It is clear from this visualization that most inconsistencies appear at cell borders. [file 1471-2105-14-319-S5.tiff]

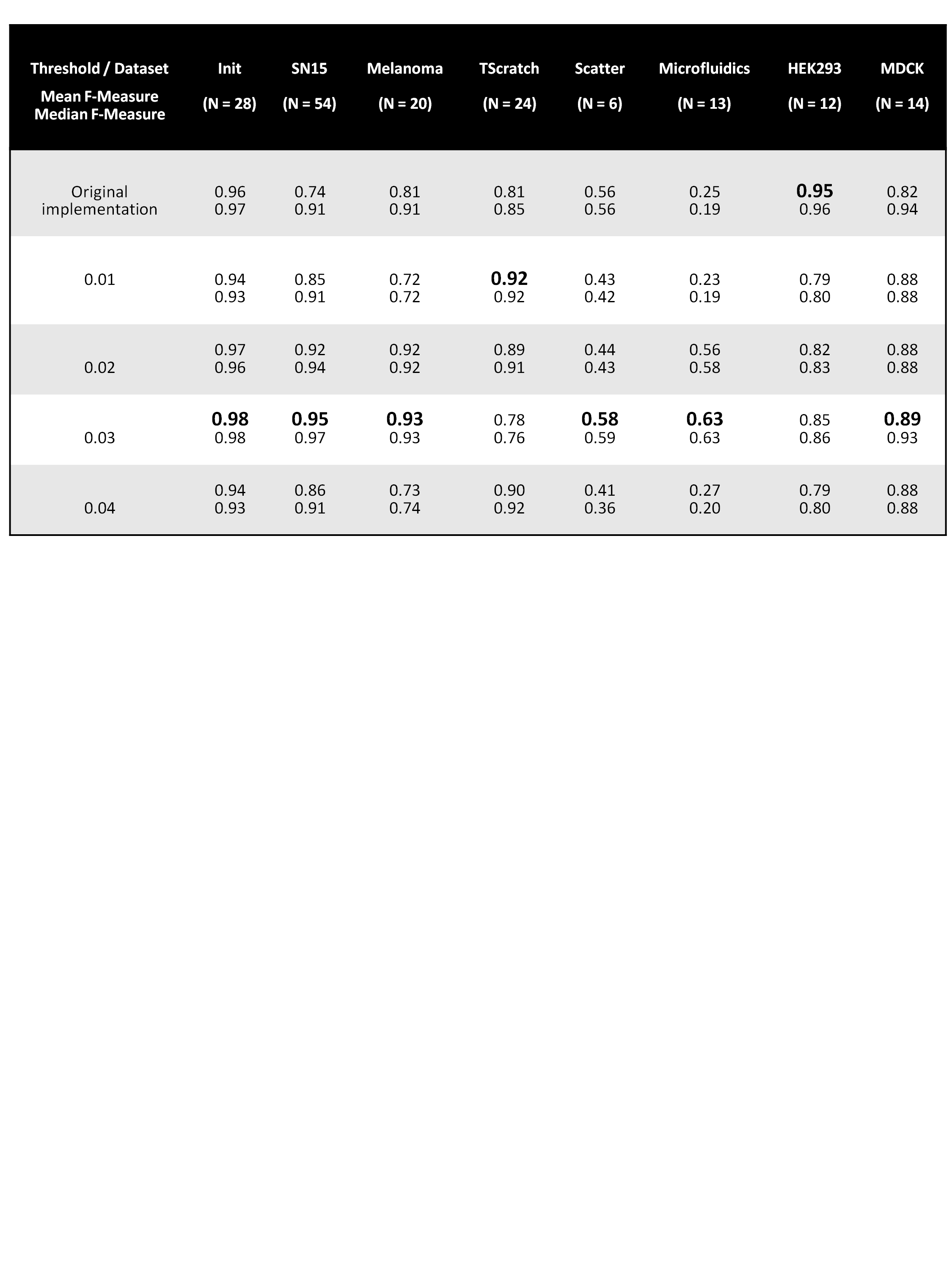

Supplement: Additional file 6: Table S3 — Adjusting Tompan’s algorithm. The automatic threshold extraction method in Topman’s algorithm was evaluated compared to a constant threshold. Evaluation of different values demonstrated that a constant threshold surpasses the automatic adjustment for most datasets. The best value found was used to evaluate this algorithm’s performance in the main text. [file 1471-2105-14-319-S6.tiff]
